# Environmental and occupational respiratory diseases – 1060. Clinical responses to methylprednisolone pulse therapy in children with severe refractory mycoplasma pneumoniae pneumonia

**DOI:** 10.1186/1939-4551-6-S1-P58

**Published:** 2013-04-23

**Authors:** Jae Ho Lee, Sun Young You, Eun Ae Yang, You Sook Youn, Hong Ryang Gil

**Affiliations:** 1 Pediatrics, Chungnam National University School of Medicine, Dae Jeon, South Korea; 2 Pediatrics, St Mary's Hospital, The Catholic University of Korea, Daejon, Korea

## Background

*Mycoplasma pneumoniae* (*M. pneumoniae*) pneumonia is one of the most common causes of community acquired pneumonia in children. The clinical course is usually self limited and benign. But rarely severe pneumonia could be complicated despite appropriate antibiotic therapy. We determine the impact of methylprednisolone pulse therapy on the severe refractory *M. pneumoniae* pneumonia in children.

## Methods

We evaluated the clinical effects of steroid pulse therapy retrospectively in 12 children with severe refractory *M. pneumoniae* pneumonia, diagnosed serologically. All of the patients, who showed the respiratory distress, high fever and initial lobar pneumonic consolidation with pleural effusion radiologically, and deteriorated despite of antibiotic therapy were treated with intravenous methylprednisolone pulse therapy in addition to antibiotics.

## Results

The average febrile period prior to admission was 4.9±1.7 days and that was 3.7±1.6 days after using antibiotics. We initiated methylprednisolone pulse therapy at a dose of 30mg/kg on the day 5.4±2.5 of admission. After pulse therapy, the clinical symptoms and signs were improved in all patients without adverse events of steroid therapy. In particular, the high fever was subsided within 0-2 hours after initiation of steroid pulse therapy. The abnormal radiologic findings were resolved on the days 2.6±1.3 and the high levels of C-reactive protein (6.7±5.9mg/dl on admission) was decreased to 1.3±1.7mg/dl on the days 3.0±1.1 of steroid therapy.

## Conclusions

This study showed an impact of 3-day methylprednisolone pulse therapy on severe refractory *M. pneumoniae* pneumonia in children despite appropriate antibiotic therapy. The steroid pulse therapy is apparently an efficacious and well-tolerated treatment for severe refractory *M. pneumoniae* pneumonia.

